# Survey of awareness and beliefs about cancer (ABC) in Tehran Province, Iran

**DOI:** 10.1186/s12885-024-12211-y

**Published:** 2024-05-11

**Authors:** Paria Akbari, Saeed Nemati, Azin Nahvijou, Paria Bolourinejad, Lindsay Forbes, Kazem Zendehdel

**Affiliations:** 1 Cancer Research Center, Cancer Institute, Tehran University of Medical Sciences, Tehran, Iran; 2 Students’ Scientific Research Center, Tehran University of Medical Sciences, Tehran, Iran; 3 Student Research Committee, School of Medicine, Isfahan University of Medical Science, Isfahan, Iran; 4 Centre for Health Services Studies, University of Kent, Canterbury, UK

## Abstract

**Introduction:**

Knowledge, attitudes, and practices are essential measures for planning and evaluating cancer control programs. Little is known about these in Iran.

**Methods:**

We conducted a population-based interview survey of adults aged 30–70 using the Farsi version of the Awareness and Beliefs about Cancer questionnaire in the capital province of Tehran, Iran, 2019. We calculated weighted estimates of levels of cancer knowledge, attitudes, and practices to allow for different selection probabilities and nonresponse. We used multivariate logistic regression to understand demographic factors associated with bowel, cervix, and breast screening practices.

**Results:**

We interviewed 736 men and 744 women. The mean number of recalled cancer warning signs was less than one; 57.7% could not recall any cancer warning signs. Participants recognized 5.6 out of 11 early cancer warning signs and 8.8 of 13 cancer risk factors. Most (82.7%) did not know that HPV infection was a cancer risk factor. Approximately, half had negative attitudes towards cancer treatment, but over 80% had positive attitudes towards the effectiveness of screening for improving survival. Colorectal, breast, and cervical screening rates were 24%, 42%, and 49%, respectively. Higher socioeconomic status increased the odds of taking up screening for cancer. Women aged 60–70 were less likely to report taking up breast and cervical screening than younger women.

**Discussion:**

The Iranian population has poor awareness and negative attitudes about cancer, and participation in screening programs is low. Public awareness and early detection of cancer should be promoted in Iran.

**Supplementary Information:**

The online version contains supplementary material available at 10.1186/s12885-024-12211-y.

## Introduction

 In this study, we aimed to evaluate awareness, attitude, and practices of Iranian adults in Tehran province using a standard, validated questionnaire. The burden of cancer is increasing worldwide, particularly in low and middle-income countries (LMICs), including Iran [1]. Cancer prevention and early detection programs should be prioritized as a cost-effective solution to improve patient outcomes and reduce the burden of cancer, especially in LMICs [2, 3]. The main risk factors for cancer include tobacco, alcohol, obesity, dietary factors, low physical activity, infections, ultraviolet light, and exposure to occupational and environmental hazards that are entirely or partially avoidable [4–6]. Ultimately, by raising awareness, improving attitudes, and promoting healthy practices related to cancer risk factors and warning signs, we can enhance cancer survival [7, 8]. Assessment of population awareness about cancer is a vital step for planning and monitoring cancer control programs [9]. However, such data have been collected in only a limited number of countries, mostly western European and Northern American ones. In 2013 Forbes et al. compared cancer awareness and attitudes in 6 countries, including Australia, Canada, Denmark, Norway, Sweden, and the U.K., as part of the International Cancer Benchmarking Partnership, using the Awareness and Beliefs about Cancer (ABC) questionnaire [10]. In Iran, comprehensive data that can be compared to similar studies from other countries are currently unavailable. The absence of such information poses a significant barrier to a clear understanding of the state of cancer awareness, attitudes, and practices within our population. This poses limitations in planning for improvement in these three areas.

Several reports are available about awareness and beliefs about cancer from Asian and Middle Eastern countries [11–15]. However, only a few of these studies used validated and internationally tested tools, such as the ABC or Cancer Awareness measure (CAM), to assess the knowledge, attitudes, and beliefs of people about cancer [16, 17]. In Iran, none of the cancer knowledge, attitudes, and practices (KAP) studies have been based on validated questionnaires like ABC or CAM. Additionally, the studies in Iran has been mostly focused on a particular type of cancer and assessed practices or awareness of a targeted population. Therefore, the present body of evidence fails to provide a clear, comprehensive image of cancer KAP in Iran.

To bridge this gap, we conducted a survey using the Farsi version of the international ABC questionnaire to examine the KAP among the adult population of Tehran Province. By employing the ABC questionnaire in our study, we will be able to provide information that is internationally comparable.

## Methods

### Population

Adult Iranian citizens, living in urban and rural areas of Tehran Province aged between 30 and 70 years old were the target population.

### Sampling

We used a multistage cluster random sampling method and proportion-to-size sampling scheme to select the survey subjects residing in Tehran Province. Tehran Province has 16 counties, including the capital city of Tehran and 15 other counties.

The sampling frame was a list of households held by the Iranian Students Polling Agency (ISPA). We carried out the survey in the county of Tehran city and a randomly selected 5 further counties in the province of Tehran. Each county is divided into municipality districts (Mantangheh) and districts are divided into urban neighbourhoods (Mahalleh). Primary sampling units were defined as Mahallehs. We selected all 22 districts in Tehran County and considered each of the randomly selected counties equal to a district, as districts in Tehran County are almost as highly populated as the other counties in Tehran Province. In each of the 27 districts we performed systematic sampling of PSUs, the number of PSUs in proportion to the population of the district [18]. In each PSU we randomly selected one street to sample households from. The PSU sampling was performed at the Cancer Institute of Iran, while the interviewers sampled the secondary sampling units (SSUs) i.e. households.

Trained interviewers initiated the survey by inviting the very first household located at the beginning of one side of each street, and subsequently, households were chosen at 50 household intervals. Within each SSU, the interviewer asked about the age and sex of the people present at home, and if eligible to take part in the study, a modified KISH Table [19] was employed to choose ten individuals aged between 30 and 70 years. We randomly selected household members after stratifying for sex and age group. When enough individuals from both sexes were sampled in a specific age group, it was marked as completed [20]. This was continued until participants from all age groups were interviewed in the SSUs. Because of the age distribution within households in Iran, this modification was necessary, as the WHO/KISH table could potentially lead to an underestimation of adults over 55 years old. The provided KISH table was adjusted for age and sex.

During the survey, the interviewers interviewed one participant per household, completing each cell of the sampling tables accordingly. Each sampling table was assigned to each SSU and had 10 cells to be filled: one male and one female for each of the five age groups. If a household did not include individuals within the specified age or sex group, the interviewer was instructed to approach neighbouring homes or the next apartment within the multistory buildings and follow the same sampling method until all the boxes of the sampling tables were filled.

We carefully planned the sampling method to reduce the probability of selection bias, however, most of the participants where Tehran city residents, and only a small number of study population were rural areas habitants. Therefore, our results cannot be easily applicable to all geographical areas or provinces in Iran, especially the underprivileged cities or provinces with a majority of rural population.

### Questionnaire

The Awareness and Beliefs about Cancer (ABC) questionnaire is a validated measure for assessing population awareness, attitudes and practices regarding cancer. The measure was first developed in 2012 by Simon et al. in the UK [21] for use in an international study in the UK, Canada, Australia and Scandinavia and has been translated into several other languages. The ABC questionnaire seemed to be the best instrument for our context and would allow comparison of the results with other populations. A detailed report about the translation and adaptation of the ABC questionnaire for the Farsi language has been published elsewhere [22].

In brief, we performed minor modifications and linguistic validation of the ABC questionnaire for application in awareness surveys in Iran. The main modifications were in the questions related to demographic and socioeconomic information. We also added questions regarding cervical screening attitudes and behaviours that were not included in the original questionnaire.

### Data collection

Our experienced interviewers, who were trained for this study, filled in printed questionnaires during face-to-face interviews with each participant. They obtained verbal informed consent before starting the interview, clearly explaining the goals and public benefits of this study, its implications, estimated time of the interview, and requirements of participating in this study. The content of the verbal informed consent was previously approved by the Ethical Committee of Tehran University of Medical Sciences, as no invasive process was involved in our study, participation was entirely voluntary, and no direct or indirect harm was expected for participants. Our interviewers ensured that all the participants, including the illiterate ones, absorbed all the information before giving consent for the interview. Since there are no formal or legal guardians for illiterate adults in Iran, literate, trusted adults in the households assisted the illiterate participants if required. The Ethical Committee of Tehran University of Medical Sciences approved this protocol, issuing the ethical approval code IR.TUMS.VCR.REC.1396.4518.

The data collection was performed from August to September 2018.

### Analysis

First, we calculated probability weights that were the reverse of the sum of the possibility of being selected through the three-stage sampling. Then, we computed the post-stratifications weight by dividing the sample size on population size of Tehran Province by sex and age and performed a weighted analysis in all steps, considering sampling weight and post stratification weights. Primary Sampling Units (PSUs) were considered the only clustering stages, and standard error estimation was carried out using the Taylor linearization technique [23]. Descriptive analysis was performed using means for continuous variables and proportions for categorical variables with associated 95% confidence intervals (95% CI). Finally, we used a multiple logistic regression model to study the associations between participation in breast, cervical, and colorectal screening and age group, marital status, SES, family history of cancer, and self-rated health.

To determine socioeconomic status (SES), we used level of education, living standards, and asset variables on a binary scale, including number of rooms relative to number of occupants; housing tenure; property assets; and ownership of vehicles, household appliances and electronic gadgets. These values were converted to a continuous variable using Principal Component Analysis (PCA), using the first component weight. The SES score was then categorized into quintiles of very high, high, middle, low, and very low.

## Results

We conducted a survey of 1481 Iranian adults aged 30–70 in Tehran Province, Iran using the Farsi version of the Awareness and Beliefs about Cancer questionnaire. Our researchers approached 1500 individuals for interviews and succeeded in interviewing 1481, giving a response rate of 98%. About half were men (*N* = 736). The average age was 44.2 (SD: 11.0, Min = 30, Max = 69). Around 60% had a high-school diploma or above. Approximately 20% of the study population was single, and most of them were urban residents (Table 1).

**Table 1:** Demographic characteristics of participants of the knowledge, attitude, and practice (KAP) survey in Tehran Province, Iran, in 2019

Variable	Number (%)
**Gender**	
Male	737 (49.7)
Female	744 (50.3)
**Age group**	
30–39	625 (42.2)
40–49	382 (25.7)
50–59	289 )19.5)
60–70	185 (12.4)
**Marital Status**	
Married	1196 (80.7)
Single	128 (8.6)
Divorced	22 (1.4)
Widowed	39 (2.6)
Missing	96 (6.4)
**Health Insurance**	
Yes	1195 (80.6)
No	267 (18.0)
Unknown	19 (1.2)
**Education level**	
Illiterate	68 (4.5)
Primary	234 (15.8)
Secondary	198 (13.3)
High school, no diploma	79 (5.3)
High school diploma	460 (31.0)
University	431 (29.1)
No response	11 (0.74)
**SES**^a^	
Very low	253 (20.0)
Low	252 (19.9)
Middle	252 (19.9)
High	252 (19.9)
Very high	252 (19.9)
**Family history of cancer**	
Positive	536 (36.2)
Negative	945 (63.8)
**Self-rated health**	
High	1028 (69.4)
Low	453 (30.6)
**Residence area**	
City of Tehran	994 (67.2)
Other cities in Tehran Province	413 (27.8)
Rural area in Tehran Province	74 (4.9)
**Overall**	1481 (100)^a^
^a^SES score was calculted for 1261 participants because of missing data	

### Knowledge

**Fig. 1:**
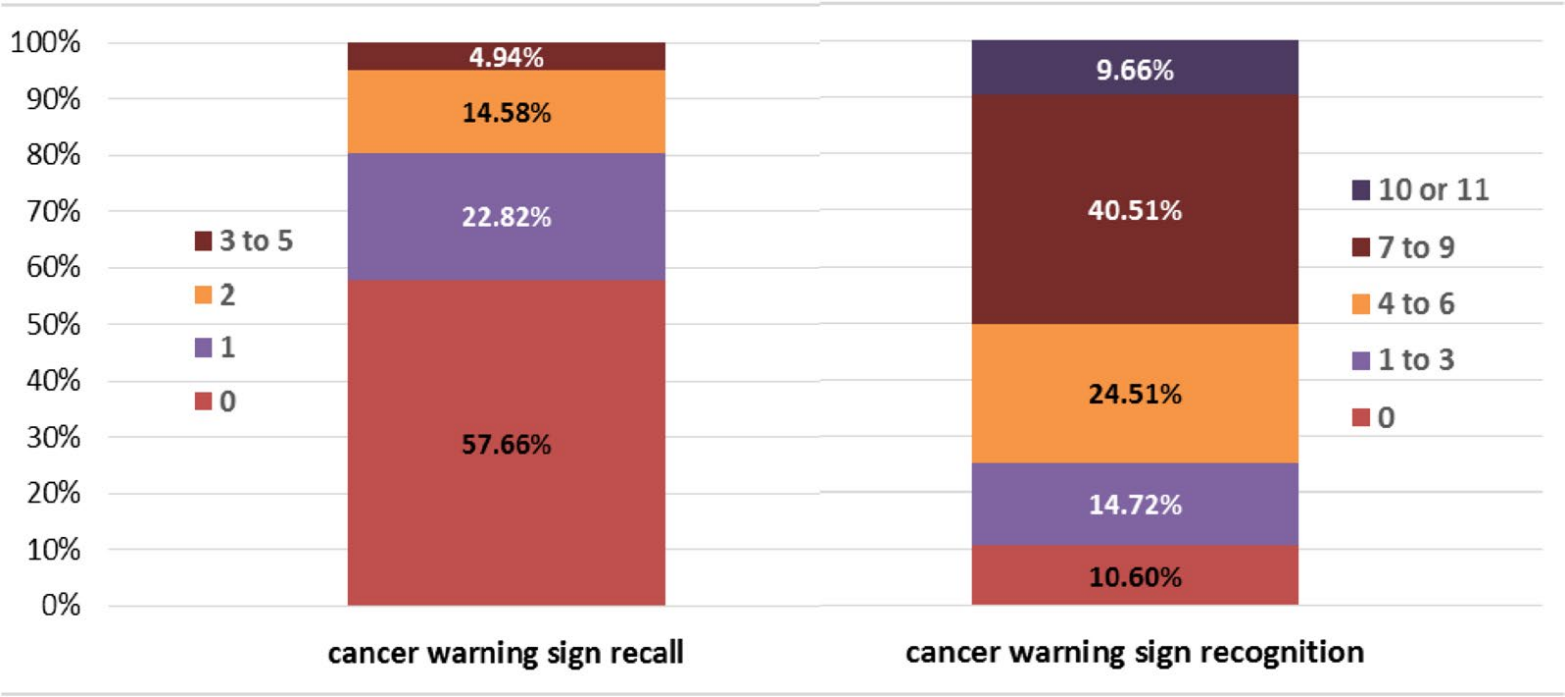
Cancer symptom recall of the general population living in Tehran Province in 2019

In our study, 57.6% were not able to specify any cancer warning signs. On average, participants recalled 0.67 warning signs. However, they recognized an average of 5.8 out of 11 warning signs from a provided list (Fig. 1). The most recognized warning signs were unexplained weight loss (57.6%), persistent unexplained pain (53.1%), unexplained bleeding (49.1%), and unexplained lump (48.2%). Less than half of the participants recognized unexpected night sweats (30.8%), persistent cough or hoarseness (35.6%), changes in bladder and bowel habits (36.1%), unexplained fatigue (37.9%), non-healing sores (38.3%), changes in the appearance of a mole (39.9%), or persistent difficulty in swallowing (41.5%) as cancer warning signs.

**Fig. 2:**
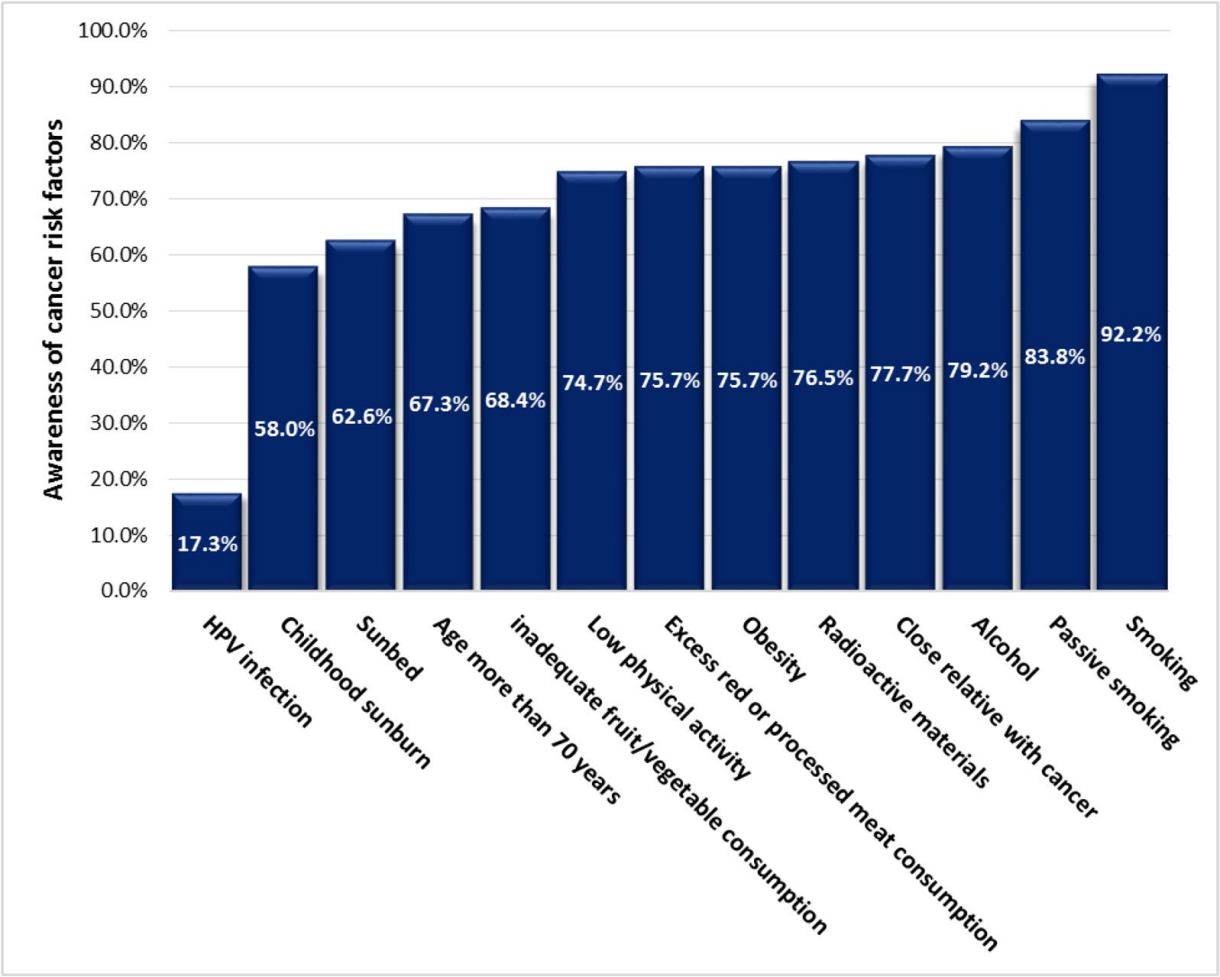
Knowledge regarding cancer risk factors in the general population living in Tehran Province in 2019

In this study, participants recognized an average of 8.8 out of 13 risk factors, and 81% of them identified at least 7 risk factors. However, 82% of the study population had never heard of the Human Papilloma Virus (HPV) infection as a risk factor for cervical cancer. Roughly one-third of the participants did not consider old age, sunburn in childhood, and unhealthy diet as risk factors for different types of cancer (Fig. 2).

### Attitudes

A substantial proportion of the study population expressed their intention to visit a doctor within two weeks upon noticing changes in their breasts (89.5% of women) or experiencing rectal bleeding (90.9%). However, the percentage of individuals reporting the same timeframe for seeking medical advice was lower for persistent cough (77.6%) and abdominal bloating (56.1%) (Data not shown). The substantial number of individuals expressing a willingness to promptly consult a physician upon observing changes in breast conditions or experiencing rectal bleeding indicates their positive attitude to seeking medical attention for potentially serious symptoms. However, lack of intention to seeing a doctor within two weeks in case of persistent cough or abdominal bloating highlights the concerns about health-seeking behaviors and perceived severity of symptoms.

Regarding beliefs about cancer, more than half of Tehran province residents (58.3%) believed that the complications of cancer treatments would be worse than the disease itself (Table 2). This promotes concerns on understanding of people about the impact of cancer treatments on quality of life. Additionally, 30.8% of participants indicated a preference for not being informed if they were diagnosed with cancer, emphasizing on the concerns about emotional burden of cancer diagnosis. Approximately half of the respondents (53.7%) considered a cancer diagnosis to be a death sentence, which might affect psychological well-being and treatment preferences. Furthermore, 61.1%, 61.7%, and 58.3% of the participants believed that cancer screening is necessary only when they experienced symptoms of breast, bowel, and cervical cancer, respectively. This result indicates that people do not realise that screening is intended for people who are not symptomatic, either for early detection at a pre-symptomatic stage or for prevention.

Most of the respondents stated that they would not avoid breast (83.2%), bowel (81.6%), and cervical (76.7%) screening tests due to fear of the possible results, indicating an overall positive attitude towards cancer prevention and screening. More than 80% of the participants believed in the effectiveness of screening tests in reducing cancer mortality. Around two-thirds of the sample (66.3%) believed that there is a high chance of curing cancer, and a similar percentage (64.6%) believed that cancer patients can continue their normal daily activities. Moreover, 82.5% of the respondents believed that visiting a doctor promptly after experiencing cancer symptoms would improve their chances of survival (Table 2).

**Table 2:** Beliefs about cancer in a general adult population living in Tehran Province, Iran, in 2019

Beliefs regarding cancer screening and prognosis			Disagree %	Agree %
Negative Beliefs	Most cancer treatment is worse than cancer itself		41.69	58.31
	I would not want to know if I had cancer		69.16	30.84
	A diagnosis of cancer is a death sentence		46.22	53.78
	Cancer screening is only necessary if I have symptoms	Breast	38.90	61.1
		Bowel	38.29	61.71
		Cervical	41.62	58.38
	I would be so worried about what might be found at cancer screening that I would prefer not to do it	Breast	83.22	16.78
		Bowel	81.63	18.37
		Cervical	76.76	23.23
Positive Beliefs	Cancer screening could reduce my chance of dying from cancer	Breast	13.08	86.92
		Bowel	13.86	86.15
		Cervical	13.07	86.93
	These days, many people with cancer can expect to continue with normal activities and responsibilities		35.30	64.7
	Cancer can often be cured		33.68	66.32
	Visiting a doctor as quickly as possible after noticing a symptom of cancer could increase the chances of surviving		17.48	82.53

### Practices

**Fig. 3:**
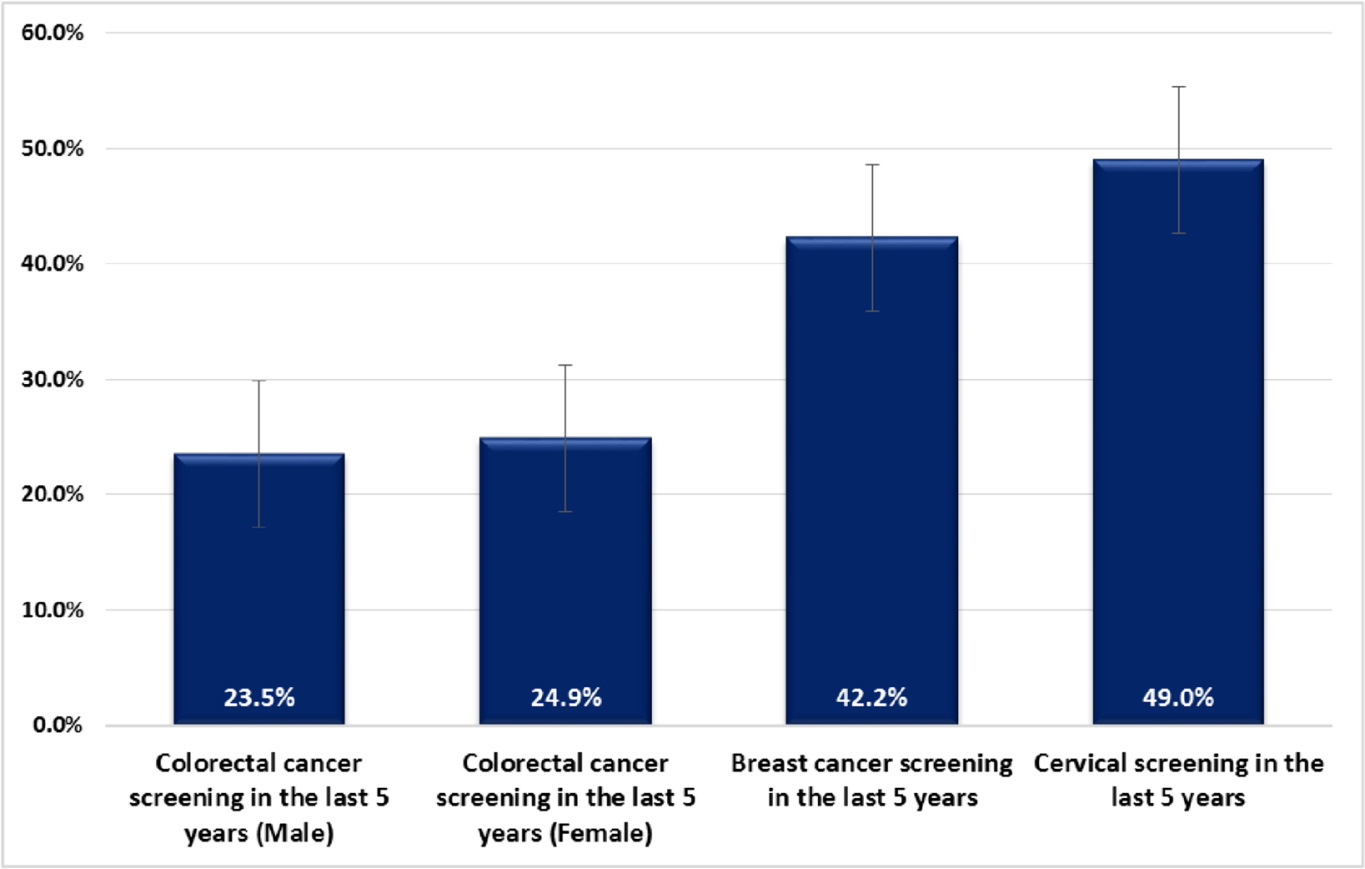
Prevalence of colorectal, breast, and cervical cancer screening in the general population living in Tehran Province (2019) in women, and men. (Note: for colorectal and breast screening, the analysis was restricted to people older than 50 years)

A faecal occult blood test for bowel cancer screening during the previous five years was reported by 23% of men and 25% of women. Nearly half of the women had had cervical screening and 42% reported having had mammography for breast cancer screening in the previous five years (Fig. 3). Compared with the reference group, women 60–70 years old were less likely to report having had a breast screening (OR = 0.68, 95% CI: 0.31, 1.48) or cervical screening (OR = 0.43, 95% CI: 0.2–0.91) (Table 3). In men older than 60, reporting having had bowel cancer screening was significantly higher than in the reference 50–59 years age group. (OR = 2.37, 95% CI: 1.07–5.22). People in the highest SES groups were more likely to have participated in bowel screening (OR = 2.27 male, 1.66 female) and women in the highest SES groups were significantly more likely to have participated in cervical screening (OR = 2.92, 95% CI: 1.65–5.17) (Fig. 3).

**Table 3:** Adjusted odds ratios (ORs) and 95% confidence intervals (95% CIs) for different factors associated with recent colorectal, breast and cervical screening participation in Tehran Province in 2019

Variable^a^		Colorectal screening				Breast mammography screening		Cervical screening	
		Male		Female					
	No. of Screened (%)	Adjusted OR (95%CI)^b^	No. (%)	Adjusted OR (95%CI)^b^	No. (%)	No. of Screened (%)	Adjusted OR (95%CI)^b^	No. of Screened (%)	OR (95%CI)
**Age group**									
30–39	Excluded^c^	Not relevant	Excluded^c^	Not relevant	Excluded^c^	Excluded^c^	Not relevant	160(21.8)	Reference
40–49	Excluded^c^	Not relevant	Excluded^c^	Not relevant	Excluded^c^	Excluded^c^	Not relevant	96(14.2)	0.99 (0.66, 1.46)
50–59	66(15.2)	Reference	27(13.1)	Reference	39(17.3)	69(32.3)	Reference	75(10.4)	1.22 (0.79, 1.88)
60–70	49(9)	**2.37 (1.07, 5.22)**	32(10.4)	1.17 (0.47, 2.91)	17(7.6)	20(9.9)	0.68 (0.31, 1.48)	19(2.6)	**0.43 (0.2, 0.91)**
**Family history**									
Yes	51(27.5)	Reference	22(25.1)	Reference	29(29.8)	51(51.1)	Reference	161(54.4)	Reference
No	64(22.2)	1.03 (0.48, 2.18)	37(22.5)	0.83 (0.37, 1.85)	27(21.9)	38(35.6)	0.54 (0.27, 1.05)	188(45.6)	0.81 (0.56, 1.17)
**Marital status**									
Married	93(23.9)	Reference	50(22.7)	Reference	43(25)	67(41.2)	Reference	299(50.9)	Reference
Single	0(0)	-----	0(0)	-----	0(0)	2(38.2)	0.56 (0.07, 4.41)	10(28.6)	**0.2 (0.07, 0.53)**
Other	22(31.5)	1.03 (0.33, 3.21)	9(36)	0.95 (0.39, 2.33)	13(27.3)	20(46.3)	1.47 (0.69, 3.14)	41(50)	0.64 (0.35, 1.18)
**SES (Socioeconomic status)**									
Lowest	19(20.8)	Reference	10(20.9)	Reference	9(20.7)	12(31.6)	Reference	48(41.7)	Reference
Low	21(19.2)	0.74 (0.25, 2.15)	10(14.4)	1.16 (0.39, 3.38)	11(23.8)	20(44.7)	1.59 (0.68, 3.73)	62(50.3)	1.52 (0.87, 2.65)
Middle	17(25.7)	0.89 (0.25, 3.09)	6(17)	1.89 (0.57, 6.23)	11(34)	17(55.9)	2.19 (0.85, 5.61)	58(52.8)	1.71 (0.91, 3.19)
High	21(30.1)	1.78 (1.03, 5.41)	13(36.4)	1.29 (0.41, 4.02)	8(24.1)	14(42.2)	1.4 (0.55, 3.52)	63(49.7)	1.52 (0.91, 2.52)
Highest	17(33.8)	2.27 (0.65, 7.94)	12(35.3)	1.66 (0.40, 6.78)	5(32.3)	11(68.6)	**3.62 (1.02, 12.74)**	69(61.8)	**2.92 (1.65, 5.17)**
**Self-rated health**									
High	78(28.1)	Reference	38(25.1)	Reference	40(31)	61(48.1)	Reference	242(49.6)	Reference
Low	35(19)	0.76 (0.34, 1.72)	20(20.5)	0.56 (0.26, 1.23)	15(17.5)	27(34.4)	0.58 (0.29, 1.15)	105(50.2)	1.09 (0.7, 1.68)
Total	115(24.2)		59(23.5)		59(23.6)	89(42.3)		350(49.22)	

^a^for colorectal and breast screening, all analyses were restricted to people older than 50 years

^b^Adjusted for all variables

^c^Not recommended for age less than 50 years

## Discussion

We conducted a survey in Tehran Province and found that cancer KAP were low. The majority of participants were not aware of cancer warning signs. In addition, a large proportion of participants were not aware of the impact of HPV infection on developing cervical cancer. More than half believed that cancer treatments were worse than cancer itself, that a cancer diagnosis is a death sentence, and that screening is required only after the onset of symptoms. Less than 50% of participants had been screened for common malignancies in recent years, which was lower than the rate of screening in most of the western countries [24]. Notably, we observed a positive association between SES and participation in screening. Evidence from various studies indicated the same relationship between getting screened for cancer and SES [25]. People older than 59 years were more likely to do a bowel cancer screening test, while women of 60 years age or older had lower odds of being screened for cervical and breast cancer.

The main limitation of this survey is that we included Tehran Province residents only, and most of the participants were urban residents. According to the results from the last national census in 2016, educational level is considerably higher in Tehran Province compared to most of the other provinces in Iran [26]. This is also true for the SES of Tehran population. Therefore, we cannot generalise the results to the whole Iranian population, especially to people living in less urban areas.

In the present study, the number of correctly identified early cancer symptoms was lower than studies in developed countries, but similar to the findings of a Malaysian study. Participants in surveys in the UK, Denmark, Norway, Sweden, Australia, USA, and Canada recognized on average 8–9 cancer symptoms [10, 27, 28]. The number of cancer symptoms recognized by Malaysians was 5.8 [16]. The average number of recalled warning signs in our study was 0.67, which was close to the result of similar studies in Malaysia and other Asian countries. A multicenter study in the Asian Pacific region showed that 30% of participants could not recall any colorectal cancer warning sign [29] and in a study in Malaysia, the population recalled a mean of 0.2 early cancer symptoms [16]. Only 48.2% of our participants considered a lump or swelling as a warning sign, whereas this symptom was recognized by 74.5% of participants from Malaysia [16] and more than 90% of responders from Denmark [27], Sweden [30], UK [31], and USA [28]. Instead, weight loss and pain occurring without any identified reason were the two most-recognized early cancer symptoms in our study. Changes in the appearance of a mole was another highly recognized symptom in studies of western countries (more than 90%) but under-recognized in the present study (around 40%).

The mean number of risk factors recognized by participants in our study was 8.8 out of 13, which was better than that in Malaysia (7.5 out of 12) [16] and close to that in Denmark (9 out of 13) [27] and Canada (9 out of 11) [32]. As in many other countries, smoking was the most recognized (92.2%) cancer risk factor in Iran, followed by alcohol (79.2%). The rate of awareness about smoking as a risk factor for cancer was over 95% in Sweden, Canada, Denmark, Saudi Arabia, and 88.7% in Malaysia. Low intake of fruit and vegetables was identified by nearly 70% of our study population as a cancer risk factor, which was worse than the results in Canada (84%) but higher than the awareness in Saudi Arabia (58%), Malaysia (52%) and Denmark (41%). In Iran and Malaysia, people were less aware of sunburn in childhood as a cancer risk factor compared to the population in Denmark, Sweden, and Canada. Iranians had lower awareness rate about HPV (17.3%) being a cancer risk factor compared to people in other countries Like Canada (85.56%), Saudi Arabia (59%), and Malaysia (52.8%), and close to Denmark (23%) [16, 27, 32–34].

The percentage of participants who believed that cancer treatments are worse than the cancer itself (58.3%) was close to the same proportion in the USA (59.6%) and Denmark (59.6%), and less than Korea (79.9%) [35–37]. About two thirds of Iranians in this study believed that cancer could often be cured and people with cancer would be able to continue their normal daily routine, which was lower than in people living in high income countries like UK, Western Europe, USA, Korea, Australia and Canada [10, 35–37]. Positive feelings about prognosis after being diagnosed with cancer were reported among approximately half of Iranians and Koreans, but among more than 70% of people in Europe, Australia, and USA [10, 35, 36]. Around 10% of the study population in the UK and Denmark, 23% of them in the USA, nearly half of them in Korea, and one third of them in Iran claimed that they do not wish to know if they have been diagnosed with cancer [35–38]. These negative beliefs and concerns might deter people from starting and adhering to cancer treatment plans.

In our study, 82.5% of participants agreed that early help seeking in case of any cancer warning sign might reduce mortality. In comparison, more than 90% in the UK, Denmark, USA, and Korea believed so. Considering the common belief among our participants that they should only take up cancer screening if they are symptomatic and they would not want to know about their possible cancer diagnosis, there is a high risk they would miss the opportunity of early diagnosis. The relatively high percentage of the participants who were unaware of the importance of cancer screening suggests that there should be further information campaigns about screening. Since people are not invited routinely for cancer screening in Iran, individuals need advice about its benefits and the appropriate frequency of testing.

It is evident that people tend to take rectal bleeding and breast changes more seriously than other cancer warning signs. Whereas about 90% of our study population anticipated visiting a doctor in less than two weeks if they felt any changes in their breasts or observed rectal bleeding, they were less likely to do so in case of persistent cough (77.6%) or bloating (56.1%). At least 90% of participants in the UK, Spain, Malaysia, Denmark and Sweden mentioned that they would see a doctor in less than two weeks in case of rectal bleeding or changes in breasts. However, only around half of the participants in the UK, Denmark and Sweden, more than two third of Spanish people, and approximately 80% in Malaysia claimed that they would go to a doctor within two weeks for a persistent cough [31, 39–41].

We found out that screening tests for most prevalent cancers were not taken up by the majority of participants, which could be partly due to a lack of access to organized screening programs in Iran. While opportunistic screening for breast and cervix has been available for a long time in Iran, colorectal screening has only been introduced recently [42]. Less than 30% of the study population had faecal occult blood test and 42 and 49% of women had undergone mammography and cervical screening, respectively. In comparison, less than 10% of the study populations in India, Pakistan, and Malaysia and around 15% of the Turkish population had undergone colorectal cancer screening, and nearly half of the population in Australia had been screened for colorectal cancer. The rate for breast cancer and cervical screening in Iran is higher than Turkey and Malaysia, and close to the rates in Australia [15, 29, 43–46].

The odds of having taken up breast cancer (OR = 0.68) and cervical (OR = 0.43) screening were significantly lower in women aged 60–70 years than younger women. Lower screening rates in older age groups have also been reported in previous studies [47], and this should be considered for interventions to increase cancer screening. Older women should be the focus of campaigns for promoting cancer screening, particularly regarding breast cancer screening, with cervical cancer being less common after the age of 64. It is not surprising that higher participation was linked to higher SES in our study, because it is more affordable and may be more accessible to this group.

The KAP score would likely be lower in less developed areas compared to larger and more developed cities. The state of cancer knowledge and attitudes among less-educated people with low SES, especially in remote rural parts of the country, might be worse than those in our study population [48, 49]. Participation in screening among residents of small, remote cities and villages might be affected by limited access and poor infrastructure, low insurance coverage, inappropriate transportation, lack of health care facilities for early detection of cancers and insufficient human resources, including gastroenterologists, pathologists, gynecologists, radiologists, etc. It would be informative to repeat this study in a different part of Iran and compare the results in different sub-populations. This would help us to better understand the differences between provinces with various SES and cultural backgrounds.

To summarize, in a well-designed survey, we found that awareness and practice of the Iranian population about cancer risk factors and early detection is not satisfactory, particularly regarding HPV infection. The enormous gap in knowledge about HPV should be addressed in future interventions and studies in Iran. Improving awareness about HPV and encouraging regular cervical screening is essential for reducing the incidence rate (2.3 per 100,000) and mortality rate (1.5 per 100,000) of cervical cancer among Iranian women [50]. We suggest using the ABC questionnaires and conducting similar surveys at the national level in Iran and the international level in low and middle income countries. The results of this study can be used as the basis for population awareness about cancer and launch health promotion programs to improve cancer prevention measures in Iran. In addition, governments should consider the results of such studies as key indicators for evaluating national cancer control programs and repeat such surveys regularly over time.

### Supplementary Information

**Supplementary Material 1.**

**Supplementary Material 2.**

## Data Availability

The datasets used or analysed during the current study available from the corresponding author upon reasonable request.
