# Supplementary material for: Survey of awareness and beliefs about cancer (ABC) in Tehran Province, Iran
Source: BMC Cancer. 2024 May 11;24:579. doi: 10.1186/s12885-024-12211-y (PMC11088007; doi:10.1186/s12885-024-12211-y)
Supplement: Supplementary file 1 — Supplementary Material 1. [file 12885_2024_12211_MOESM1_ESM.pdf]

Ipsos MORI

Awareness and Beliefs about Cancer (ABC) measure

Final version in UK English

COUNTRY OF ORIGIN AUTOMATICALLY SET BY CATI SCRIPT

IF APPOINTMENT PREVIOUSLY MADE

Please can I speak to [INSERT NAME FROM APPOINTMENT LOG]?

INTRODUCTION

Good morning / afternoon / evening, my name is <<(INSERT)>> and I work for the independent research agency Ipsos MORI. We are carrying out a health research survey on behalf of Cancer Research UK and would like to invite you, or a member of your household, to take part.

This is a national survey of people from across England who are aged 50 or over and the survey is also being carried out in 7 other countries.

READ OUT ONLY IF ASKED: The participating countries are England, Northern Ireland, Wales, Norway, Sweden, Denmark, Australia and Canada.

READ OUT TO ALL: The results of the survey will be used to explore differences in people's views about cancer across different countries and to help improve cancer information for the general public. It will also help doctors to diagnose cancer earlier.

ASK ALL

SINGLE CODE

ALLOW "REFUSED" OR "DON'T KNOW" BUT THANK AND CLOSE IF THEY DO REFUSE OR DON'T KNOW

QS1. This survey only covers people aged 50 or older. Could you please tell me how many people aged 50 or over currently live in your household, including yourself?

01	One	CONTINUE TO INFORMATION SECTION
02	Two	ASK QS2
03	Three	ASK QS3
04	Four	ASK QS3
05	Five	ASK QS3
06	Six	ASK QS3
07	Seven	ASK QS3
08	Eight	ASK QS3
09	Nine	ASK QS3
10	Ten	ASK QS3
11	None	THANK AND CLOSE

IF CODE 2 AT QS1: THE CATI SCRIPT WILL SELECT ONE ADULT RANDOMLY: CATI WILL SELECT THE CURRENT RESPONDENT ON 50% OF OCCASIONS, AND THE OTHER HOUSEHOLD MEMBER ON 50% OF OCCASIONS. IF THE CURRENT RESPONDENT IS SELECTED, CONTINUE TO INFORMATION SECTION. IF OTHER HOUSEHOLD MEMBER SELECTED ASK

SINGLE CODE

ALLOW "REFUSED" BUT THANK AND CLOSE IF THEY DO REFUSE

QS2. In households where there are two adults aged 50 or older, we are using a random method to select which one of these adults takes part in this survey. On this occasion, it is the other person that I would like to speak to. May I speak to that person please?

READ OUT ONLY IF ASKED: This is to ensure we achieve a nationally representative sample of adults in England.

01 Yes, available **REREAD INTRODUCTION** AND CONTINUE TO INFORMATION SECTION

02 Yes, not available MAKE APPOINTMENT, AND LOG NAME OF SELECTED HOUSEHOLD MEMBER

03 No THANK AND CLOSE

IF CODES 3-10 AT S1: THE CATI SCRIPT WILL SELECT ONE ADULT RANDOMLY – THE PROBABILITY OF SELECTION IS LINKED TO THE NUMBER OF HOUSEHOLD MEMBERS AT S1. CATI WILL SELECT THE CURRENT RESPONDENT IN (1/S1) OCCASIONS. IT SHOULD SELECT THE OTHER HOUSEHOLD MEMBERS IN ((S1-1)/S1) OCCASIONS. IF THE CURRENT RESPONDENT IS SELECTED, CONTINUE TO INFORMATION SECTION. IF OTHER HOUSEHOLD MEMBER SELECTED ASK

SINGLE CODE

ALLOW "REFUSED" BUT THANK AND CLOSE IF THEY DO REFUSE

QS3. Where there are three or more adults aged 50 or older, we are using a random method to select one person to interview. Not including yourself, please could I speak to the person aged 50 or over who has the next birthday?

INTERVIEWER NOTE: THIS DOES NOT INCLUDE THE PERSON YOU ARE SPEAKING TO, IT MUST BE ANOTHER MEMBER OF THE HOUSEHOLD.

READ OUT ONLY IF ASKED: The person with the next birthday is selected to ensure we achieve a nationally representative sample of adults in England.

01 Yes, available **REREAD INTRODUCTION** AND CONTINUE

02 Yes, not available MAKE APPOINTMENT, AND LOG NAME OF SELECTED HOUSEHOLD MEMBER

03 No THANK AND CLOSE

04 I don't know who has the next birthday THANK AND CLOSE

INFORMATION

If you do decide to take part, the survey would take around 15-20 minutes to complete. All information that you give us will be treated in the strictest confidence and your identity will not be passed on to a third party.

Your details will not be passed on to your GP or doctor. Whether or not you decide to take part, this will not affect your health care in any way.

READ OUT ONLY IF ASKED: Your telephone number has been randomly generated. These numbers are not obtained from any commercially available calling list. Using this process we do not know any details about the household we are calling.

READ OUT ONLY IF ASKED: Ipsos MORI is a member of the Market Research Society and your personal data will be held in accordance with the Data Protection Act

VOLUNTARY NATURE OF THE SURVEY

It is up to you to decide whether or not to take part. If you decide to take part you are still free to stop at any time and without giving a reason. If you prefer, you can also skip individual questions on the survey.

ASK ALL

SINGLE CODE

QS4. Now that I have told you about the survey, would you be willing to take part?

01	Yes	CONTINUE
02	No – not available right now	MAKE APPOINTMENT TO CALL BACK AND LOG NAME
03	No – do not want to take part	GO TO QS5

ASK ALL WHO SAY 'NO' AT QS4

MULTICODE

ALLOW "REFUSED"

ENQUIRE GENTLY WITHOUT INSISTING ON AN ANSWER. DO NOT READ OUT

QS5. Please don't feel you have to say, but would you be willing to tell me why you don't want to be interviewed, just to help us get a general idea of why people aren't taking part?

01	It would be upsetting / uncomfortable / emotionally difficult to take part
02	I don't have time
03	Questionnaire is too long
04	I don't take part in surveys
05	I'm not interested
06	I don't know anything about cancer
07	Have personal experience of cancer so would be upsetting to take part
08	Other

END SURVEY

FOR RESPONDENTS WHO APPEAR DISTRESSED AT ANY POINT OR WHO HAVE CONCERNS OR QUESTIONS ABOUT CANCER – OFFER TO END THE TELEPHONE CALL AND OFFER CONTACT DETAILS AS REQUIRED:

TO SPEAK TO A CANCER NURSE, PLEASE CALL CANCER RESEARCH UK'S FREEPHONE HELPLINE 0808 800 4040 (FREEPHONE NUMBER; MON-FRI 9am-5pm)

IF YOU HAVE BEEN AFFECTED BY CANCER, CALL THE MACMILLAN CANCER SUPPORT LINE: 0808 808 0000 (FREEPHONE NUMBER; MON-FRI 9am-8pm)

SAMARITANS: 08457 90 90 90 (NOT FREE BUT OPERATES 24 HOURS A DAY)

Ipsos MORI

Awareness and Beliefs about Cancer (ABC) measure

Interview questionnaire

DEMOGRAPHIC / BACKGROUND INFORMATION 1

I would now like to ask you a couple of questions about yourself, which will help us to analyse the results of the survey.

ASK ALL

WRITE IN

ALLOW "REFUSED"

Q1. What was your age last birthday? RECORD EXACT AGE

IF REFUSED PROBE: Which age group applies to you?

SINGLE CODE. READ OUT

ALLOW "REFUSED"

01 50-54

02 55-59

03 60-64

04 65-69

05 70-74

06 75-79

07 80-84

08 85-89

09 90+

ASK ALL

SINGLE CODE

Q2. INTERVIEWER TO CODE GENDER

01 Female

02 Male

ASK ALL

SINGLE CODE

ALLOW “REFUSED” OR “DON’T KNOW”

Q3. Have you, or any friends or family members that are close to you, ever been diagnosed with cancer?

IF ‘YES’, PROBE: May I ask, is that you, someone close to you or both you and someone close to you?

01 Yes, respondent (self)

02 Yes, someone close

03 Yes, both self and someone close

04 Yes, but would prefer not to say who

05 No

CANCER AWARENESS

This question is about your awareness of, and beliefs about, cancer; it is not assessing your personal risk of cancer. This is not a test, we are interested in your thoughts and beliefs so please answer the questions as honestly as you can.

READ OUT ONLY IF ASKED: I am sorry that I can't answer detailed questions during the interview but we can go back to these at the end if you like. But I won't be able to go back at the end to change any answers.

WARNING SIGNS/SYMPTOMS

ASK ALL

WRITE IN

ALLOW "DON'T KNOW" OR "REFUSED"

Q4. There are many warning signs and symptoms of cancer. Please name as many as you can think of.

READ OUT ONLY IF ASKED: Please think about all different types of cancer.

RECORD ALL OF THE WARNING SIGNS OR SYMPTOMS THAT THE PERSON MENTIONS EXACTLY AS THEY SAY IT AND PROMPT UNTIL THE RESPONDENT CANNOT THINK OF ANY MORE SIGNS: Can you think of any others?

01 ……………………………………………………………………………………

02 ……………………………………………………………………………………

03 ……………………………………………………………………………………

04 ……………………………………………………………………………………

05 ……………………………………………………………………………………

06 ……………………………………………………………………………………

07 ……………………………………………………………………………………

08 ……………………………………………………………………………………

09 ……………………………………………………………………………………

10 ……………………………………………………………………………………

ANTICIPATED DELAY IN SEEKING MEDICAL HELP

ASK ALL

SINGLE CODE FOR EACH PART. ROTATE QUESTIONS Q5-Q8

ALLOW “DON’T KNOW” OR “REFUSED”

The next questions are about going to the doctor. I’m going to read you out a list of signs and symptoms. For each one please tell me how long it would take you to go to the doctors from the time you first noticed the symptom.

Q5. A persistent cough?

READ OUT ONLY IF ASKED: By persistent I mean that it has lasted for some time.

Q6. Rectal bleeding, that is bleeding from the back passage or blood in the bowel motions?

ASK WOMEN ONLY

Q7. Any breast changes?

ASK ALL

Q8. Abdominal bloating? By abdominal, I mean your tummy or belly.

IF WOULD NOT GO TO DOCTOR PROBE FULLY FOR REASON

01 Up to 1 week

02 Over 1 up to 2 weeks

03 Over 2 up to 3 weeks

04 Over 3 up to 4 weeks

05 More than a month

06 I would go as soon as I noticed

07 I would not contact my doctor

08 I would go to a pharmacist instead of a doctor:

09 I would go to a nurse (at my GP surgery) instead of a doctor:

10 I would go to a healthcare professional at an NHS Walk In Centre instead of a doctor

11 I would go to a healthcare professional at a hospital instead of a doctor

I'm now going to list some symptoms that may or may not be warning signs for cancer. For each one, can you tell me whether you think that it could be a warning sign for cancer?

ASK ALL

SINGLE CODE FOR EACH PART. ROTATE QUESTIONS Q9-Q19

ALLOW "REFUSED" AND "DON'T KNOW"

Q9-19. Do you think [INSERT WARNING SIGN] could be a sign of cancer?

READ OUT ONLY IF ASKED: By persistent I mean that it has lasted for 3-6 weeks.

READ OUT ONLY IF ASKED: By unexplained I mean that it is not due to an illness or injury that you already know about.

READ OUT ONLY IF ASKED: By a change in bowel and bladder habits I mean a change in pooing and weeing.

READ OUT ONLY IF ASKED: By night sweats I mean sweats that wake you and make your sheets damp

Q9. an unexplained lump or swelling[a]

Q10. a persistent unexplained pain

Q11. unexplained bleeding

Q12. a persistent cough or hoarseness

Q13. a change in bowel or bladder habits

Q14. a persistent difficulty in swallowing

Q15. a change in the appearance of a mole[b]

Q16. a sore that does not heal

Q17. unexplained night sweats

Q18. unexplained weight loss

Q19. unexplained tiredness

01	Yes
02	No

[a] This item had low discrimination (>95% respond 'yes')

[b] This item had low discrimination (>95% respond 'yes')

SELF-RATED HEALTH, ACCESS TO A DOCTOR AND SMOKING

I would just like to ask you a couple more questions about yourself.

ASK ALL

SINGLE CODE. READ OUT

ALLOW "DON'T KNOW" OR "REFUSED"

ROTATE RESPONSE OPTIONS FOR 50% OF RESPONDENTS

Q20. In general, would you say your health is…?

01 Very good
02 Good
03 Fair
04 Poor
05 Very poor

ASK ALL

SINGLE CODE. READ OUT

ALLOW "DON'T KNOW" OR "REFUSED"

ROTATE RESPONSE OPTIONS FOR 50% OF RESPONDENTS

Q21. How easy, or difficult, is it for you to get to see a doctor if you have a symptom that you think might be serious?

01 Very difficult
02 Somewhat difficult
03 Somewhat easy
04 Very easy

ASK ALL

SINGLE CODE

ALLOW "DON'T KNOW" OR "REFUSED"

Q22. Do you smoke at all these days, either cigarettes, including hand-rolled ones, pipes or cigars?

01 Yes
02 No

ASK ALL WHO SAY 'NO' AT Q22

SINGLE CODE

ALLOW "DON'T KNOW" OR "REFUSED"

Q23. Have you ever smoked either cigarettes, including hand-rolled ones, pipes or cigars?

01 Yes
02 No

ACCESS TO CARE: EARLY SYMPTOMATIC PRESENTATION

Sometimes people put off going to see the doctor even when they have a symptom they think might be serious. These are some of the reasons people give for delaying. Could you say if any of these might put you off going to the doctor?[c]

For each one that I read out, please respond either 'Yes, often', 'Yes, sometimes', or 'No'.

ASK ALL

SINGLE CODE FOR EACH PART. ROTATE QUESTIONS Q24-Q27

IF RESPONDENT REQUESTS, READ OUT RESPONSES AGAIN

ALLOW "DON'T KNOW" OR "REFUSED"

Q24. I would be too embarrassed.

Q25. I would be worried about wasting the doctor's time.

Q26. I would be worried about what the doctor might find.

Q27. I am too busy to make time to go to the doctor.

01	Yes, often
02	Yes, sometimes
03	No

[c]'Barriers to symptomatic presentation' aggregate variable uses Qs24-27

GENERAL CANCER BELIEFS AND BELIEFS ABOUT EARLY SYMPTOMATIC PRESENTATION AND EARLY DIAGNOSIS OF CANCER

I'm now going to read you some statements that are sometimes made about cancer.

For each of the statements can you tell me how much you agree or disagree with each item?[d]

ASK ALL

SINGLE CODE FOR EACH PART. ROTATE QUESTIONS Q28-Q32

ALLOW "DON'T KNOW" OR "REFUSED"

IF RESPONDENT SAYS AGREE / DISAGREE: Is that strongly or tend to agree / disagree?

Q28. These days, many people with cancer can expect to continue with normal activities and responsibilities.

Q29. Most cancer treatment is worse than the cancer itself.

Q30. I would NOT want to know if I have cancer.

Q31. Cancer can often be cured.

Q32. Going to the doctor as quickly as possible after noticing a symptom of cancer could increase the chances of surviving.

01	Strongly disagree
02	Tend to disagree
03	Tend to agree
04	Strongly agree

ASK ALL

SINGLE CODE

ALLOW "DON'T KNOW" OR "REFUSED"

Q33. Some people think that a diagnosis of cancer is a death sentence. To what extent do you agree or disagree that a diagnosis of cancer is a death sentence?

IF RESPONDENT SAYS AGREE / DISAGREE: Is that strongly or tend to agree / disagree?

01	Strongly disagree
02	Tend to disagree
03	Tend to agree
04	Strongly agree

[d] 'Beliefs about cancer outcomes' aggregate variable using Q28, Q31 and Q33

ASK ALL

WRITE IN NUMBER (FROM 0-10) FOR EACH PART. ROTATE QUESTIONS Q34-Q37

ALLOW "DON'T KNOW" OR "REFUSED"

I would now like you to think about people with different types of cancer and how long they may live after finding out they have cancer.[e]

Q34. Out of 10 people diagnosed with bowel cancer, how many do you think would be alive 5 years later?

Q35. Out of 10 people diagnosed with breast cancer, how many do you think would be alive 5 years later?

Q36. Out of 10 people diagnosed with ovarian cancer, how many do you think would be alive 5 years later?

Q37. Out of 10 people diagnosed with lung cancer, how many do you think would be alive 5 years later?

01 Record number of people here …………

RISK

And I would now like you to think about the population in general.

ASK ALL

SINGLE CODE. READ OUT

ALLOW "DON'T KNOW" OR "REFUSED"

Q38. Over the next year, which of these groups of people do you think is most likely to be diagnosed with cancer?

01 30 year olds

02 50 year olds

03 70 year olds

04 People of any age are equally likely to be diagnosed with cancer

[e] Qs 34-37 had a high number of 'invalid' responses indicating that participants found them difficult to answer.

Module 1 Cancer Screening beliefs and behaviour

CANCER SCREENING BEHAVIOUR

The next section is about cancer screening programmes that invite everyone in a particular age group to have tests to check whether they may have bowel or breast cancer.

Firstly I am interested in whether you have attended screening.

ASK ALL FEMALES

SINGLE CODE.

ALLOW "DON'T KNOW", "REFUSED" OR "NOT APPLICABLE"

QM1. Have you had a breast cancer screening test, mammogram, in the past 5 years?

01 Yes

02 No

ASK ALL

SINGLE CODE.

ALLOW "DON'T KNOW", "REFUSED" OR "NOT APPLICABLE"

QM2. Have you had a bowel cancer screening test in the past 5 years?

01 Yes

02 No

BELIEFS ABOUT CANCER SCREENING

ASK ALL FEMALES

SINGLE CODE FOR EACH PART. ROTATE QUESTIONS QM3-QM5

ALLOW "DON'T KNOW" OR "REFUSED"

The next items are about breast cancer screening, mammograms. Can you tell me how much you agree or disagree with each item?

IF RESPONDENT SAYS AGREE / DISAGREE: Is that strongly or tend to agree / disagree

QM3. I would be so worried about what might be found at breast cancer screening that I would prefer not to have it

QM4 Breast cancer screening is only necessary if I have symptoms

QM5 Breast cancer screening could reduce my chance of dying from breast cancer.[f]

01 Strongly disagree

02 Tend to disagree

03 Tend to agree

04 Strongly agree

Now I'm going to ask you some items about bowel cancer screening.

For each of the statements can you tell me how much you agree or disagree with each item?

[f] This item had low test-retest reliability, k=0.11

ASK ALL

SINGLE CODE FOR EACH PART. ROTATE QUESTIONS QM6-QM8

ALLOW “DON’T KNOW” OR “REFUSED”

IF RESPONDENT SAYS AGREE / DISAGREE: Is that strongly or tend to agree / disagree?

QM6. I would be so worried about what might be found at bowel cancer screening, that I would prefer not to do it.

QM7 Bowel cancer screening is only necessary if I have symptoms

QM8 Bowel cancer screening could reduce my chances of dying from bowel cancer.

01 Strongly disagree

02 Tend to disagree

03 Tend to agree

04 Strongly agree

Ipsos MORI

Module 2 Cancer risk factor awareness

I am now going to read out a list of things which may or may not increase your chances of getting cancer in general.

For each one can you tell me how much you agree or disagree that it may increase your chances of getting cancer?

ASK ALL

SINGLE CODE FOR EACH PART. ROTATE QUESTIONS QN1-QN13

ALLOW “DON’T KNOW” OR “REFUSED”

IF RESPONDENT SAYS AGREE / DISAGREE: Is that strongly or tend to agree / disagree?

QN1. Smoking?[g]

QN2. Exposure to another person’s smoke?

QN3. Drinking more than 1 unit of alcohol a day. One unit of alcohol is equivalent to a single measure of spirits, a third of a pint of normal strength lager or beer, or a small glass of wine?

QN4. Eating less than 5 portions of fruit and vegetables a day?

QN5. Eating red or processed meat once a day or more? By processed meat I mean meat which is smoked, salted or chemically preserved.

QN6. Being obese?

QN7. Getting sunburnt more than once as a child?

QN8. Being over 70 years old?

QN9. Having a close relative with cancer?

QN10. Infection with HPV, Human Papillomavirus?

QN11. Not doing much physical activity?

QN12. Using a sunbed?[h]

QN13. Exposure to radiation such as radioactive materials, x-rays or radon

01	Strongly disagree
02	Tend to disagree
03	Tend to agree
04	Strongly agree
05	I don’t know what this is (FOR QN10 ONLY)

---

[g] This item had low test-retest reliability, k=-0.02

[h] This item had low test-retest reliability, k=-0.04

DEMOGRAPHIC / BACKGROUND INFORMATION 2

I would now like to ask you a few more questions about yourself, to help us analyse the results of the survey.

ASK ALL

SINGLE CODE. READ OUT

ALLOW "REFUSED / PREFER NOT TO SAY"

Q39ENG Which of these best describes your ethnic group?

White

01 English / Welsh / Scottish / Northern Irish / British

02 Irish

03 Gypsy or Irish Traveller

04 Any other White background

Mixed / multiple ethnic groups

05 White and Black Caribbean

06 White and Black African

07 White and Asian

08 Any other Mixed / multiple ethnic background

Asian / Asian British

09 Indian

10 Pakistani

11 Bangladeshi

12 Chinese

13 Any other Asian background

Black / African / Caribbean / Black British

14 African

15 Caribbean

16 Any other Black/ African / Caribbean background

Other ethnic group

17 Arab

199 Any other ethnic group (SPECIFY)

ASK ALL WHO SAY 'OTHER' AT Q39ENG

WRITE IN

ALLOW "REFUSED"

Q39oENG. Could you please tell me what your other ethnic group is?

ASK ALL

SINGLE CODE

ALLOW "REFUSED"

Q40. What is the main language spoken in your home?

01 English

50 Other (please specify) ……………………………

ASK ALL WHO SAY 'OTHER' AT Q40

WRITE IN

ALLOW "REFUSED"

Q40o. Could you please tell me what that language is?

ASK ALL

SINGLE CODE. READ OUT

ALLOW "REFUSED / PREFER NOT TO SAY"

Q41. What is the highest level of education you have achieved?

01 Finished school at or before the age of fifteen

02 Completed CSEs, O-levels or equivalent

03 Completed A Levels or equivalent

04 Completed further education but not a degree

05 Completed a Bachelor's degree / Masters degree / PHD

99 Other (please specify) ……………………………

ASK ALL WHO SAY 'OTHER' AT Q41

WRITE IN

ALLOW "REFUSED"

Q41o. Could you please tell me what this other education level is?

ASK ALL

SINGLE CODE. READ OUT

ALLOW “REFUSED” OR “DON’T KNOW”

Q42. Which of these best describes your current marital status?

01 Married or in a civil partnership

02 Living with my partner

03 Single, that is never married and not living with a partner

04 Divorced or separated and not living with another partner

05 Widowed and not living with another partner

ASK ALL

WRITE IN

ALLOW “REFUSED” OR "DON'T KNOW"

QPC. Could you please tell me what your postcode is? The only reason we are collecting this information is so that we can analyse the results by area, it will not be used for any other purpose.

ASK IF REFUSED AT QPC

WRITE IN

ALLOW “REFUSED” OR "DON'T KNOW"

QPC2. Would you be willing to tell me the first part of your postcode please?

AFTER THE INTERVIEW IS FINISHED

That is the end of the survey, thank you very much for your time.

ASK ALL

SINGLE CODE

QRC1. Would you be willing for Cancer Research UK to re-contact you for further research on this subject at some time in the next 12 months?

01 Yes, would be willing

02 No, would not be willing

ASK IF CODE 1 AT QRC1

WRITE IN

QRC2. Thank you very much. Please could I take your name for our records?

ASK IF CODE 1 AT QRC1

WRITE IN

QRC3. Is this the best telephone number to reach you on [INSERT NUMBER]?

01 Yes

02 No

ASK IF CODE 2 AT QRC3

WRITE IN

QRC4. Please could I take the best telephone number to call you on for our records?

FOR RESPONDENTS WHO WOULD LIKE FURTHER INFORMATION ABOUT THE SURVEY

Please contact the research team using the following details:

Colin Gardiner – 0207 347 3024 or Laura Davies – 0207 347 3323

THANK RESPONDENT AND CLOSE

Thank you on behalf of Ipsos MORI.

If you have any queries regarding the survey or our company, I can give you the telephone number of the company or the Market Research Society Freephone number.

PROVIDE AS NECESSARY

Company number – 0131 561 4603

MRS Freephone number – 0500 39 69 99

Job number – 11-000902-02

INTERVIEWERS: MRS CAN ONLY PROVIDE CONFIRMATION THAT WE ARE A GENUINE MARKET RESEARCH COMPANY
