# Supplementary material for: Survey of awareness and beliefs about cancer (ABC) in Tehran Province, Iran
Source: BMC Cancer. 2024 May 11;24:579. doi: 10.1186/s12885-024-12211-y (PMC11088007; doi:10.1186/s12885-024-12211-y)
Supplement: Supplementary file 2 — Supplementary Material 2. [file 12885_2024_12211_MOESM2_ESM.pdf]

شرکت کننده ی گرامی،
با سلام، موضوع بیماری سرطان از اهمیت و حساسیت بالایی برخوردار است. به همین منظور پرسشنامه ی حاضر تنظیم شده و در اختیار شما قرار گرفته است. بدون شک نظرات و ایده های شما در تصمیم گیری کارشناسان موثر خواهد بود. بدین منظور از شما شهروند گرامی تقاضا می شود با پاسخگویی دقیق به این پرسشنامه ما را در این مسیر یاری نمایید.این مطالعه تحت نظر مرکز تحقیقات سرطان انستیتو کنسر بیمارستان امام خمینی طراحی و اجرا شده است.
مرکز افکارسنجی دانشجویان ایران (ایسپا)

1- آیا تا به حال شما یا یکی از دوستان نزدیک یا اعضای خانواده شما، به سرطان مبتلا شده است؟ لطفا بفرمایید که آن فرد شما هستید یا فردی نزدیک به شما یا این که هر دو؟
☐1. بله، خودم ☐2. بله، یک شخص نزدیک به من ☐3. بله، هم خودم و هم یک شخص نزدیک به من
☐ 4. بله، اما ترجیح می‌دهم نگویم چه کسی
☐ 5. خیر

2- تعداد زیادی علامت و نشانه برای بیماری سرطان وجود دارد. لطفاً هر چندتا که به ذهنتان می‌رسد ذکر کنید.
☐1.................................. ☐5..................................
☐2.................................. ☐6..................................
☐3.................................. ☐7..................................
☐4.................................. ☐8..................................

3- لطفاً بگویید اگر برای اولین بار متوجه علامت سرفه مداوم شوید، حدس می‌زنید چه مدت زمان طول می‌کشد تا به پزشک مراجعه کنید؟
☐1. حداکثر تا یک هفته ☐2. یک الی دو هفته ☐3. دو الی سه هفته ☐4. سه الی چهار هفته ☐5. بیش از یک ماه
☐6. به محض این که متوجه شوم، مراجعه می‌کنم
☐7. به جای پزشك به يك داروساز مراجعه می‌كنم
☐8. به پرستار(در مطب دکتر عمومی) به جای پزشک مراجعه میکنم
☐9. به جای پزشك عمومی، به يك متخصص مراقبت سلامت در بیمارستان مراجعه می‌كنم.
☐10. با پزشکم تماس نمی‌گیرم ☐ 11. نمی‌دانم

4- لطفاً بگویید اگر برای اولین بار متوجه علامت خونریزی از مقعد یا وجود خون در مدفوع شوید، حدس می‌زنید چه مدت زمان طول می‌کشد تا به پزشک مراجعه کنید؟
☐1. حداکثر تا یک هفته ☐2. یک الی دو هفته ☐3. دو الی سه هفته ☐4. سه الی چهار هفته
☐5. بیش از یک ماه ☐6. به محض این که متوجه شوم، مراجعه می‌کنم
☐7. به جای پزشك به يك داروساز مراجعه می‌كنم
☐8. به پرستار(در مطب دکتر عمومی) به جای پزشک مراجعه میکنم
☐9. به جای پزشك عمومی، به يك متخصص مراقبت سلامت در بیمارستان مراجعه می‌كنم.
☐10. با پزشکم تماس نمی‌گیرم ☐ 11. نمی‌دانم

5- لطفاً بگویید اگر برای اولین بار متوجه علامت نفخ شکم شوید، حدس می‌زنید چه مدت زمان طول می‌کشد تا به پزشک مراجعه کنید؟
☐1. حداکثر تا یک هفته ☐2. یک الی دو هفته ☐3. دو الی سه هفته ☐4. سه الی چهار هفته ☐5. بیش از یک ماه
☐6. به محض این که متوجه شوم، مراجعه می‌کنم
☐7. به جای پزشك به يك داروساز مراجعه می‌كنم
☐8. به پرستار(در مطب دکتر عمومی) به جای پزشک مراجعه میکنم
☐9. به جای پزشك عمومی، به يك متخصص مراقبت سلامت در بیمارستان مراجعه می‌كنم.
☐10. با پزشکم تماس نمی‌گیرم ☐ 11. نمی‌دانم

6- لطفاً بگویید اگر برای اولین بار متوجه هر گونه تغییر در پستان شوید، حدس می‌زنید چه مدت زمان طول می‌کشد تا به پزشک مراجعه کنید؟ (فقط خانم‌ها پاسخ دهند)
☐1. حداکثر تا یک هفته ☐2. یک الی دو هفته ☐3. دو الی سه هفته ☐4. سه الی چهار هفته
☐5. بیش از یک ماه ☐6. به محض این که متوجه شوم، مراجعه می‌کنم
☐7. به جای پزشك به يك داروساز مراجعه می‌كنم
☐8. به پرستار(در مطب دکتر عمومی) به جای پزشک مراجعه میکنم
☐9. به جای پزشك عمومی، به يك متخصص مراقبت سلامت در بیمارستان مراجعه می‌كنم.
☐10. با پزشکم تماس نمی‌گیرم ☐ 11. نمی‌دانم

7- شما فکر می‌کنید کدامیک از موارد زیر می‌تواند علامت سرطان باشد؟
☐1. یک برجستگی یا ورم بدون توضیح ☐2. یک درد مداوم بدون توضیح ☐3. خونریزی بدون توضیح ☐4. سرفه یا گرفتگی صدای دائمی
☐5. تغییر در عادات روده یا مثانه ☐6. اشکال دائمی در بلعیدن لقمه یا غذا ☐7. تغییر در ظاهر یک خال ☐8. زخمی که خوب نمی شود
☐9. تعریق شبانه بدون توضیح ☐10. کاهش وزن بدون توضیح ☐11. خستگی بدون توضیح

8- به طورکلی سلامت خود را چگونه ارزیابی می‌کنید؟
☐1. خیلی خوب ☐2. خوب ☐3. متوسط ☐4. ضعیف ☐5. خیلی ضعیف

9- هنگامی که علائمی دارید که فکر می‌کنید ممکن است جدی باشند، دسترسی به پزشک جهت معاینه چقدر برای شما آسان یا دشوار است؟
☐1. خیلی سخت ☐2. تقریباً سخت ☐3. تاحدودی آسان ☐4. خیلی آسان

10- آیا این روزها دخانیات مصرف کرده‌اید؟ هر نوع شامل انواع دست ساز، پیپ، سیگار یا قلیان؟
☐1. بله ☐2. خیر ☐3. نمی‌دانم/ بی‌پاسخ

11- آیا تا به حال سیگار، قلیان، پیپ، سیگارهای دست‌ساز را مصرف کرده‌اید؟

❐1. بله ❐2. خیر ❐3. نمی‌دانم/ بی‌پاسخ

12- گاهی مردم حتی هنگامی که علائمی دارند که فکر می‌کنند جدی است مراجعه به پزشک را عقب می‌اندازند. بعضی از دلایلی که مردم برای تاخیر خود می‌آورند، ذکر شده است. می‌توانید بگویید کدام یک از این‌ها می‌توانند باعث شوند شما مراجعه به پزشک را عقب بیاندازید؟

ردیف	دسترسی به مراقبت	بله اغلب	بله گاهی اوقات	خیر
12-1	خیلی خجالت می‌کشم.	1	2	3
12-2	می‌ترسم وقت پزشک را تلف کنم.	1	2	3
12-3	در مورد چیزی که احتمال دارد پزشک متوجه شود نگران می‌شوم	1	2	3
12-4	من خیلی مشغله دارم و فرصت کافی برای مراجعه ندارم	1	2	3

13- اکنون برای شما جملاتی را می خوانم که گاهی در مورد سرطان گفته می شود. لطفا میزان موافقت یا مخالفت خود را با هرکدام از جملات بگویید.

ردیف	باورهای عمومی درباره سرطان	کاملاً مخالفم	تقریباً مخالفم	تقریباً موافقم	کاملاً موافقم
13-1	امروزه، خیلی از افراد مبتلا به سرطان می توانند فعالیت ها و مسئولیت های معمولی خود را ادامه دهند.	1	2	3	4
13-2	اغلبِ درمان های سرطان، از خود بیماری سرطان بدتر هستند.	1	2	3	4
13-3	اگر سرطان داشته باشم، نمی خواهم این موضوع را بدانم.	1	2	3	4
13-4	سرطان اغلب درمان می شود.	1	2	3	4
13-5	در صورتی که بلافاصله بعد از مشاهده ی علائم سرطان به پزشک مراجعه کنیم، شانس بقا و نجات از بیماری بیشتر می شود.	1	2	3	4
13-6	بعضی از مردم فکر می کنند تشخیص سرطان برابر مرگ است.	1	2	3	4

14- به نظر شما،از هر 10 نفری که برای آن‌ها هر یک از سرطان‌ها تشخیص داده می‌شود، فکر می‌کنید چند نفر بعد از 5 سال زنده خواهند ماند؟

ردیف	نوع سرطان	افرادی که بعد از 5 سال زنده می‌مانند
14-1	سرطان روده ی بزرگ	1........... نفر 2.نمیدانم❐
14-2	سرطان پستان	1........... نفر 2.نمیدانم❐
14-3	سرطان تخمدان	1...........نفر 2.نمیدانم❐
14-4	سرطان ریه	1........... نفر 2.نمیدانم❐
14-5	سرطان معده	1........... نفر 2.نمیدانم❐

15- در طول یک سال آینده فکر می کنید در کدام یک از گروه های سنی زیر احتمال تشخیص سرطان بیشتر است؟

❐1. سی ساله ❐2. پنجاه ساله ❐3. هفتاد ساله ❐4. احتمال تشخیص سرطان برای همه سنین یکسان است

16- لطفا به سوالات مربوط به باورها و رفتارهای مرتبط با غربالگری(آزمایش) سرطان پاسخ دهید.

ردیف	باورها و رفتارهای مرتبط با غربالگری سرطان	بله	خیر
16-1	آیا شما در طول یکسال گذشته تست غربالگری سرطان روده ی بزرگ (آزمایش خون در مدفوع) انجام داده‌اید؟	1	2
16-2	آیا شما در طول 5 سال گذشته یک تست غربالگری سرطان پستان (ماموگرافی) داشته‌اید؟ ☜ (فقط خانم ها پاسخ دهند)	1	2
16-3	آیا شما در طول 5 سال گذشته تست غربالگری سرطان دهانه رحم (پاپ اسمیر) انجام داده اید؟ ☜ (فقط خانم ها پاسخ دهند)	1	2

17- حال می‌خواهم درمورد غربالگری(آزمایش) سرطان روده ی بزرگ از شما سوال کنم. برای هرکدام از جملات بگویید چقدر مخالف یا موافق هستید.

ردیف	غربالگری سرطان روده ی بزرگ	کاملاً مخالفم	تقریباً مخالفم	تقریباً موافقم	کاملاً موافقم
17-1	من در مورد این که چه چیزی امکان دارد در غربالگری(آزمایش) سرطان روده ی بزرگ یافت شود، خیلی نگران می‌شوم. به همین خاطر ترجیح می‌دهم این تست را انجام ندهم.	1	2	3	4
17-2	غربالگری(آزمایش) سرطان روده ی بزرگ تنها هنگامی که علائم بیماری وجود دارد ضروری است.	1	2	3	4

17-3	غربالگری(آزمایش) سرطان روده ی بزرگ احتمال مرگ ناشی از سرطان روده بزرگ را برای من کم می‌کند.	1	2	3	4

18- حال می‌خواهم درمورد غربالگری(آزمایش) سرطان پستان و دهانه رحم از شما سوال کنم. برای هرکدام از جملات بگویید چقدر مخالف یا موافق هستید.☜ (فقط خانم ها پاسخ دهند)

ردیف	غربالگری سرطان پستان و دهانه رحم (پاپ اسمیر)	کاملاً مخالفم	تقریباً مخالفم	تقریباً موافقم	کاملاً موافقم
1-18	من در مورد این که چه چیزی امکان دارد در غربالگری سرطان پستان(ماموگرافی) یافت شود، خیلی نگران می‌شوم. به همین خاطر ترجیح می‌دهم این تست را انجام ندهم.	1	2	3	4
2-18	غربالگری سرطان پستان(ماموگرافی)تنها هنگامی که علائم وجود دارد ضروری است.	1	2	3	4
3-18	غربالگری سرطان پستان(ماموگرافی)احتمال مرگ ناشی از سرطان پستان را برای من کم می‌کند.	1	2	3	4
4-18	من در مورد این که چه چیزی امکان دارد در غربالگری سرطان دهانه رحم(پاپ اسمیر) یافت شود، خیلی نگران می‌شوم. از این‌رو ترجیح می‌دهم این تست را انجام ندهم.	1	2	3	4
5-18	غربالگری سرطان دهانه رحم(پاپ اسمیر)تنها هنگامی که علائم وجود دارد ضروری است.	1	2	3	4
6-18	غربالگری سرطان دهانه رحم(پاپ اسمیر)احتمال مرگ ناشی از سرطان دهانه رحم را برای من کم می‌کند	1	2	3	4

19- چقدر با این جمله موافق یا مخالف هستید؟ عفونت با HPV(ویروس پاپیلوم انسانی) می‌تواند خطر ابتلا به سرطان را افزایش دهد.

❒1. کاملاً مخالفم ❒2. تقریباً مخالفم ❒3. تقریباً موافقم ❒4. کاملاً موافقم ❒5. در این مورد اطلاعی ندارم

20- چقدر با این جمله موافق یا مخالف هستید؟ استفاده از دستگاه سولاریوم برای برنزه کردن می تواند خطر ابتلا به سرطان را افزایش دهد.

❒1. کاملاً مخالفم ❒2. تقریباً مخالفم ❒3. تقریباً موافقم ❒4. کاملاً موافقم ❒5. در این مورد اطلاعی ندارم

21- چقدر با این جمله موافق یا مخالف هستید؟ تماس با تشعشعات مواد رادیواکتیو، اشعه X یا رادون می تواند خطر ابتلا به سرطان را افزایش دهد.

❒1. کاملاً مخالفم ❒2. تقریباً مخالفم ❒3. تقریباً موافقم ❒4. کاملاً موافقم ❒5. در این مورد اطلاعی ندارم

22- در مورد هر کدام از مواردی که می‌خوانم، می توانید بگویید چقدر موافق یا مخالف این موضوع هستید که آن عامل می‌تواند خطر ابتلا به سرطان را افزایش دهد؟

ردیف	موارد	کاملاً مخالفم	تقریباً مخالفم	تقریباً موافقم	کاملاً موافقم	نمیدانم
22-1	سیگارکشیدن می تواند خطر ابتلا به سرطان را افزایش دهد.	1	2	3	4	5
22-2	در تماس بودن با دود سیگار شخص دیگر می تواند خطر ابتلا به سرطان را افزایش دهد.	1	2	3	4	5
22-3	نوشیدن بیش از 1 واحد الکل(تقریبا نصف یک لیوان معمولی) در روز می تواند خطر ابتلا به سرطان را افزایش دهد.	1	2	3	4	5
22-4	مصرف کمتر از 5 واحد(حدود 400 گرم) میوه یا سبزیجات در روز می تواند خطر ابتلا به سرطان را افزایش دهد.	1	2	3	4	5
22-5	مصرف گوشت قرمز یا گوشت فرآوری شده یکبار یا بیشتر در روز می تواند خطر ابتلا به سرطان را افزایش دهد.	1	2	3	4	5
22-6	چاقی می تواند خطر ابتلا به سرطان را افزایش دهد.	1	2	3	4	5

22-7	آفتاب سوختگی بیش از یکبار در کودکی می تواند خطر ابتلا به سرطان را افزایش دهد.	1	2	3	4	5
22-8	سن بالای 70 سال می تواند خطر ابتلا به سرطان را افزایش دهد.	1	2	3	4	5
22-9	داشتن یک فامیل نزدیک مبتلا به سرطان می تواند خطر ابتلا به سرطان را افزایش دهد.	1	2	3	4	5
22-10	فعالیت فیزیکی کم می تواند خطر ابتلا به سرطان را افزایش دهد.	1	2	3	4	5

اطلاعات فردی و زمینه‌ای

23- سن:..................

24- جنسیت ❑1. مرد ❑2. زن

25- میزان تحصیلات: ❑1- بی‌سواد ❑2- ابتدایی ❑3- راهنمایی ❑4- متوسطه ❑5- دیپلم ❑6- کاردانی ❑7- کارشناسی ❑8- کارشناسی ارشد ❑9- دکتری ❑10- حوزوی

26- وضعیت تأهل:

❑1- متاهل ❑2- مجرد ❑3- بدون همسر (طلاق) ❑4- بدون همسر (فوت)

27- در چه استان(ها)یی بیش از 5 سال ساکن بوده اید؟ ....

28- زبان اصلی که شما در منزل با آن صحبت می‌کنید، چیست؟

..

<u>در صورت تمایل، در مورد شغل یا حرفه‌ای که بیشترین سال‌های عمر خود را به آن اشتغال داشته‌اید، به سوالات زیر پاسخ دهید.</u>

29- عنوان شغل یا حرفه را بنویسید(……..……)

30- آیا در حال حاضر بازنشسته هستید؟ ❑1- بله ❑2- خیر

31- آیا برای سازمان یا شخص دیگری کار می‌کنید؟ ❑1- بله ❑2- خیر☜ برو به سوال 34

32- تعداد افراد شاغل در محل کار شما چند نفر است؟ .... نفر

33- آیا مسئولیتی جهت نظارت و کنترل دیگر کارمندان و کارگران به شما واگذار شده است؟ ❑1- بله ❑2- خیر

34- آیا برای انجام کار خویش افراد دیگری نیز برای شما کار می‌کنند؟ ❑1- بله

❑2- خیر☜ برو به سوال 36

35- تعداد افرادی که برای شما کار می کنند چند نفر است؟.. نفر

36- تعداد افراد ساکن این خانوار با احتساب خودتان چند نفر است؟ ....نفر

37- کدامیک از امکانات زیر در حال حاضر در خانه شما موجود است؟(شرکت کننده میتواند به چند گزینه اشاره کند یا از پاسخ دهی امتناع نماید)

❑1- ماکروویو ❑2- کامپیوتر ❑3- اتومبیل شخصی ❑4- فریزر جداگانه (سایدبای‌ساید) ❑5- تلویزیون **LED / LCD**/ پلاسما ❑6- مبل ❑7- جاروبرقی ❑8- کولر گازی یا اسپیلیت ❑9- ماشین لباسشویی تمام اتوماتیک ❑10- دسترسی به اینترنت ❑11- ماشین ظرفشویی ❑12- باغ، ویلا، زمین کشاورزی

38- کدامیک از امکانات زیر در اختیار خودتان قرار داشته و از آن استفاده می‌کنید؟(شرکت کننده میتواند به چند گزینه اشاره کند یا از پاسخ دهی امتناع نماید)

❑1- تلفن همراه ❑2- تبلت ❑3- لپ تاپ ❑4- اتومبیل شخصی ❑5- دسترسی به اینترنت

39- آیا تحت پوشش بیمه می باشید؟ ❑1- بله ❑2- خیر ❑3- عدم پاسخ‌دهی ❑4- نمی دانم

40- تحت پوشش کدام نوع بیمه می باشید؟ ❑1- بیمه سلامت ❑2- خدمات درمانی ❑3- تامین اجتماعی ❑4- نیروهای مسلح ❑5- خدمات درمانی روستایی

❑6- نامشخص ❑7- عدم پاسخ‌دهی ❑8- نمی دانم

❑9- سایر(توضیح)..

41- آیا تحت پوشش بیمه تکمیلی می‌باشید؟

❑1- بله ❑2- خیر ❑3- نمیدانم ❑4- عدم پاسخگویی

42- آیا شما مالک ساختمانی که هم اکنون در آن سکونت دارید می‌باشید، <u>به نحوی که نیاز به پرداخت هزینه نداشته باشید؟</u>

❑1- بله ❑2- خیر ❑3- نمیدانم ❑4- عدم پاسخ دهی

43- متراژ محلی که شما سکونت دارید چقدر است؟ (بدون در نظر گرفتن حیاط، مشاعات، محل نگهداری حیوانات و کارگاه)

❑ 1-.............. متر ❑2- عدم پاسخ دهی

44- تعداد اتاق‌های مسکونی خانوار خود را ذکر کنید (بدون در نظر گرفتن آشپزخانه، حمام، دستشویی و انبار).

❑1- .... ❑2- عدم پاسخ دهی

45- شماره تماس پاسخگو ....

46- منطقه.......

47- استان.......

48-شهر:............................ 49-روستا: ....

مشخصات پرسشگر ــــــــــــــــــــــــــــــ

تاریخ تکمیل پرسشنامه: ...../...../ 97 ساعت شروع مصاحبه: .... مدت زمان تکمیل پرسشنامه: .... ساعت پایان مصاحبه: ....

نام و نام‌خانوادگي پرسشگر: .... کدپرسشگر: .... کد بازبین: ....
